# Energy dashboard: post-operative insights into electrosurgical device use

**DOI:** 10.1007/s00464-025-11642-3

**Published:** 2025-03-07

**Authors:** Simon C. Baltus, Vincent J. Ribbens, Arjen Wiersma, Renske M. Hoeben, Can Ozan Tan, Ivo A. M. J. Broeders

**Affiliations:** 1 Surgery Department, Meander Medical Centre, Maatweg, 3818 TZ Amersfoort, The Netherlands; 2 Robotics and Mechatronics, University of Twente, Drienerlolaan, 5722 NB Enschede, The Netherlands

**Keywords:** Electrosurgery, Surgical training, Assessment, Fundoplication

## Abstract

**Background:**

This study presents a post-operative energy dashboard to teach surgeons about electrosurgical device use. By analyzing the energy generator, we aim to add new information to the current assessment of surgical skills. This study evaluated how such a dashboard can provide insight into differences in electrosurgery application.

**Methods:**

A semi-automated methodology for the energy dashboard was developed by acquiring intra-operative energy generator and video data, and computing metrics to compare device use. The energy dashboard quantified the use of the electrosurgical device based on the number of activations (N), the duration of individual activations (s), the total use time (s), and the total applied energy (kJ). The methodology and differences in device use were assessed based on forty-eight fundoplication surgeries performed by three surgeons.

**Results:**

The methodology identified the device activations with an F1-score of 0.95. The comparison between the surgeons showed significant differences in total usage, turn-on count, and amount of applied energy. In addition, the dashboard showed a significant difference in total applied energy (kJ) over the dissections of the gastrohepatic and gastrosplenic ligament.

**Conclusion:**

The study showed that energy monitoring can provide insights into application differences. In addition, the pilot study showed that the use of electrosurgical devices can differ significantly between surgeons. A broader application of the energy dashboard can enable a new source of information for surgical skill assessment.

Surgical skill assessment is an important part of surgical education and focuses on quantifying the technical surgical competencies [1]. Automated methods for assessing these technical skills are a growing field as they improve objectivity [2]. Currently, this field focuses on the artificial intelligence-based analysis of laparoscopic videos and kinetics [3, 4].

Electrosurgical devices are used in more than 80% of surgical procedures. The device empowers tissue dissection and hemostasis by applying energy to the tissue. However, minimizing tissue coagulation while ensuring optimal hemostasis quality remains a challenge. In addition, the technique is known for adverse events such as bleeding or damage to surrounding tissues. Therefore, skills assessment in operating electrosurgical devices is crucial [5–7].

In today’s practice, surgeons have limited insights into their performance when applying electrosurgery as this is not objectively quantified [5, 8, 9]. Monitoring the use of electrosurgical devices will provide a new source of information for surgical skills assessment. The knowledge gained provides the basis for improving device application, intending to minimize tissue coagulation and adverse events while obtaining the requested hemostasis [10].

This study focused on monitoring electrosurgical device usage by an energy dashboard. The post-operative dashboard aims to improve the device application by summarizing the analysis of the energy generator and laparoscopic video data. In addition, this study assessed the differences in device use based on fundoplication surgeries performed by different surgeons.

## Materials and methods

### Energy dashboard

**Fig. 1:**
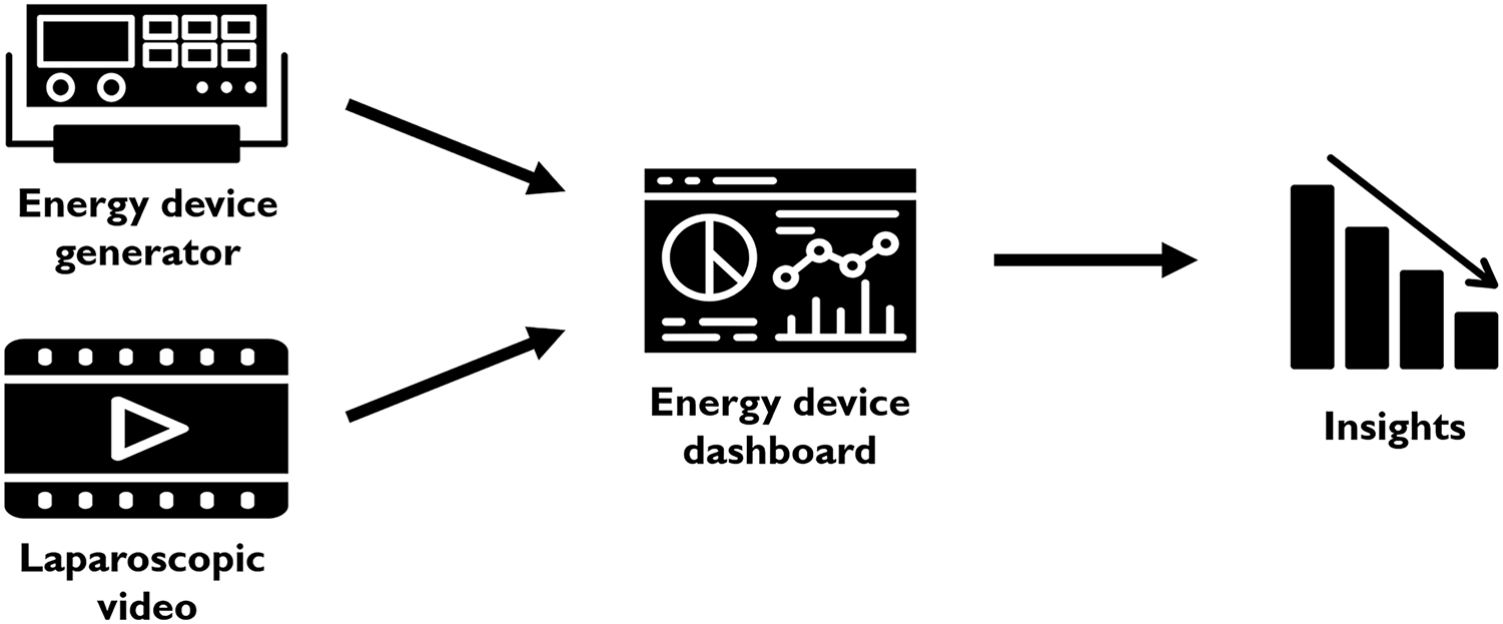
The energy device dashboard summarizes the data provided by the energy generator and the laparoscopic videos, which improves understanding and enables surgical benchmarking

The energy dashboard quantifies recent electrosurgical device use by summarizing the data from the energy generator into multiple metrics. The metrics enable the comparison between an individual’s application and those of other surgeons for each procedure. The energy data can be combined with the laparoscopic video data to analyze the metrics per surgical phase. Figure 1 provides a visualization of the concept. We developed a semi-automated methodology for this concept by acquiring multi-modal intra-operative data (1), data post-processing (2), computation of metrics (3), and hosting of dashboards (4).

**Fig. 2:**
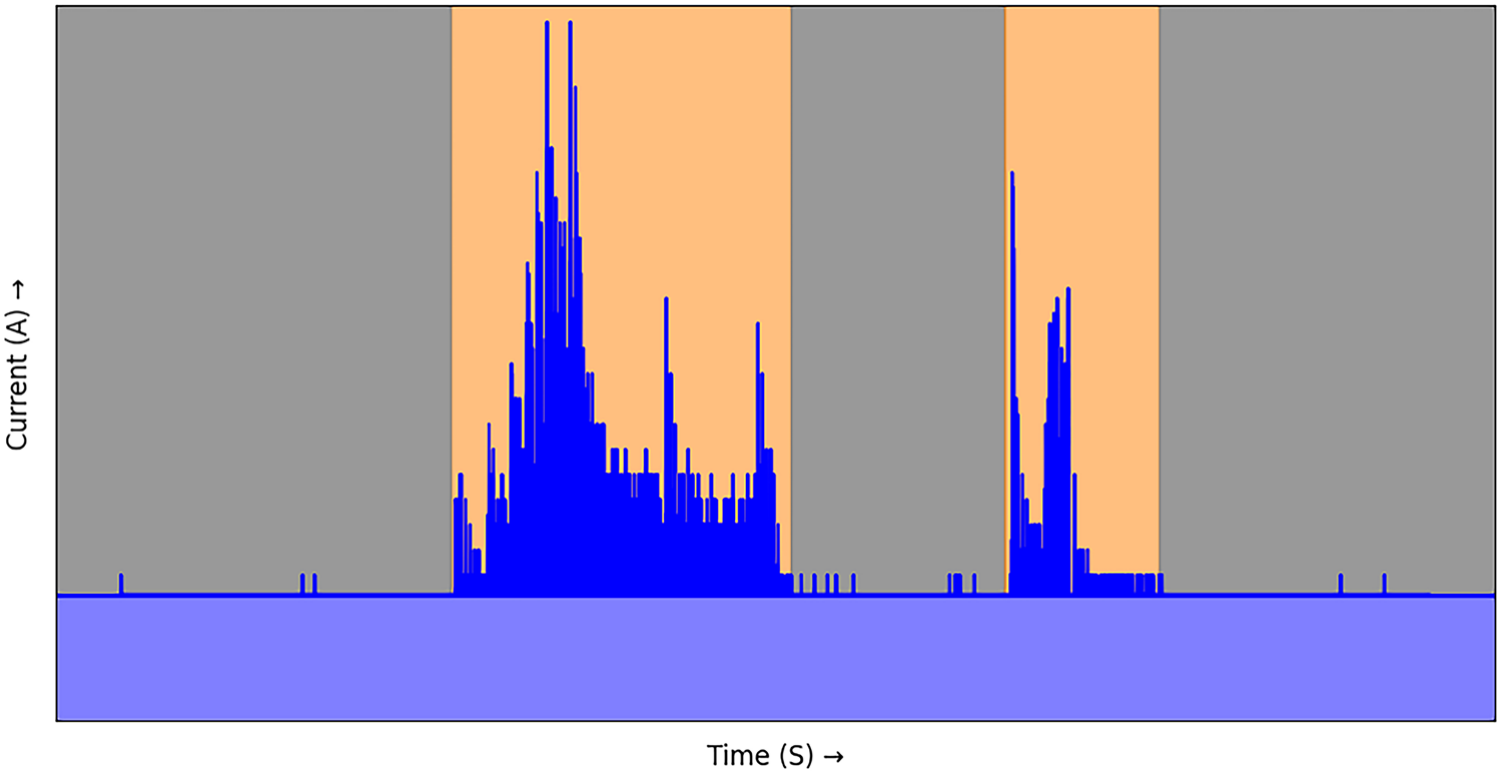
A visualization of current clamp measurement and the conversion to metrics. An activation of the device (orange) is detected as current above the baseline (light blue). Based on this, the duration and number of activations are calculated. Subsequently, the applied energy is calculated by the electrical power over time for the device activations (dark blue) (Color figure online)

1. The data acquisition for the dashboard involved recording the laparoscopic video and the current fed to the energy device’s generator (Gen11, Johnson & Johnson). The current was measured by a current clamp (Picoscope, Pico Technology) placed on the supplying power cable [11]. The clamp and corresponding oscilloscope measured the current with 500 samples per second. Both video and energy data were acquired from the beginning to the end of the surgery.
2. The acquired energy measurements were postoperatively processed using several steps. First, the current clamp measurements were compensated for a baseline current to identify the tool activations. The baseline originated from the white noise and the current used by the generator. A current period above the baseline was considered an activation of the device. Activations shorter than 0.05 s were removed. Lastly, the current was synchronized with the video based on the first visible activation in the video.
3. The application differences were quantified in the dashboard based on four metrics: number of activations (*N*), duration of individual activations (s), total use time (s), and total applied energy (kJ). The total applied energy was computed by the power (*P* = *U* × *I*) over time (*t*), which was compensated for the baseline current. The voltage (*U*) was considered consistent at 230 Volt. The total applied energy approximates the energy used but does not describe the transferred quantity. Figure 2 visualizes the computation of the metrics from the generator data.
4. Clinical implementation of the monitoring involved incorporating the metrics into a personalized dashboard using Grafana (Grafana: Open Source Data visualization). Each surgeon could enter their dashboard through an internal link address and password. The dashboard visualized a surgeon’s metrics compared to others for the same procedures.

The performance of the semi-automated activation detection was validated based on nine surgeries. In these cases, an additional recording of the generator’s audio was performed. A blinded annotator manually annotated the audio signals corresponding to the tool’s activation. A comparison between the automated detection and the manual annotations was used to quantify the performance. The F1-score measured the methodology’s performance.

### Pilot study

The differences in the application of electrosurgery were assessed in fundoplication surgeries. The surgeries were performed by three surgeons with different levels of experience. Surgeon 1 had twenty-five years of experience in diaphragmatic hernia repairs, Surgeon 2 had ten years, and Surgeon 3 had five years. Patients who underwent diaphragmatic hernia repair between May 2023 and December 2024 and had both simultaneous energy and laparoscopic video recordings were included in a prospective manner.

**Fig. 3:**
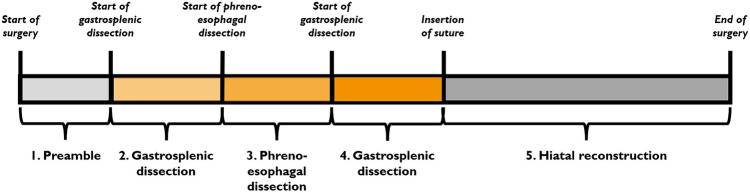
Visualization of the different phases of the diaphragmatic hernia repair

The four metrics were quantified for the total duration of the surgery and the five phases of the diaphragmatic hernia repair. The phases are defined as follows: preamble, dissection of the hepatogastric ligament, dissection of the phrenoesophageal membrane, dissection of gastrosplenic ligament, and hiatal reconstruction. The definitions of the phases are shown in Fig. 3. A physician manually annotated the phases based on the laparoscopic videos.

## Results

### Energy dashboard

**Fig. 4:**
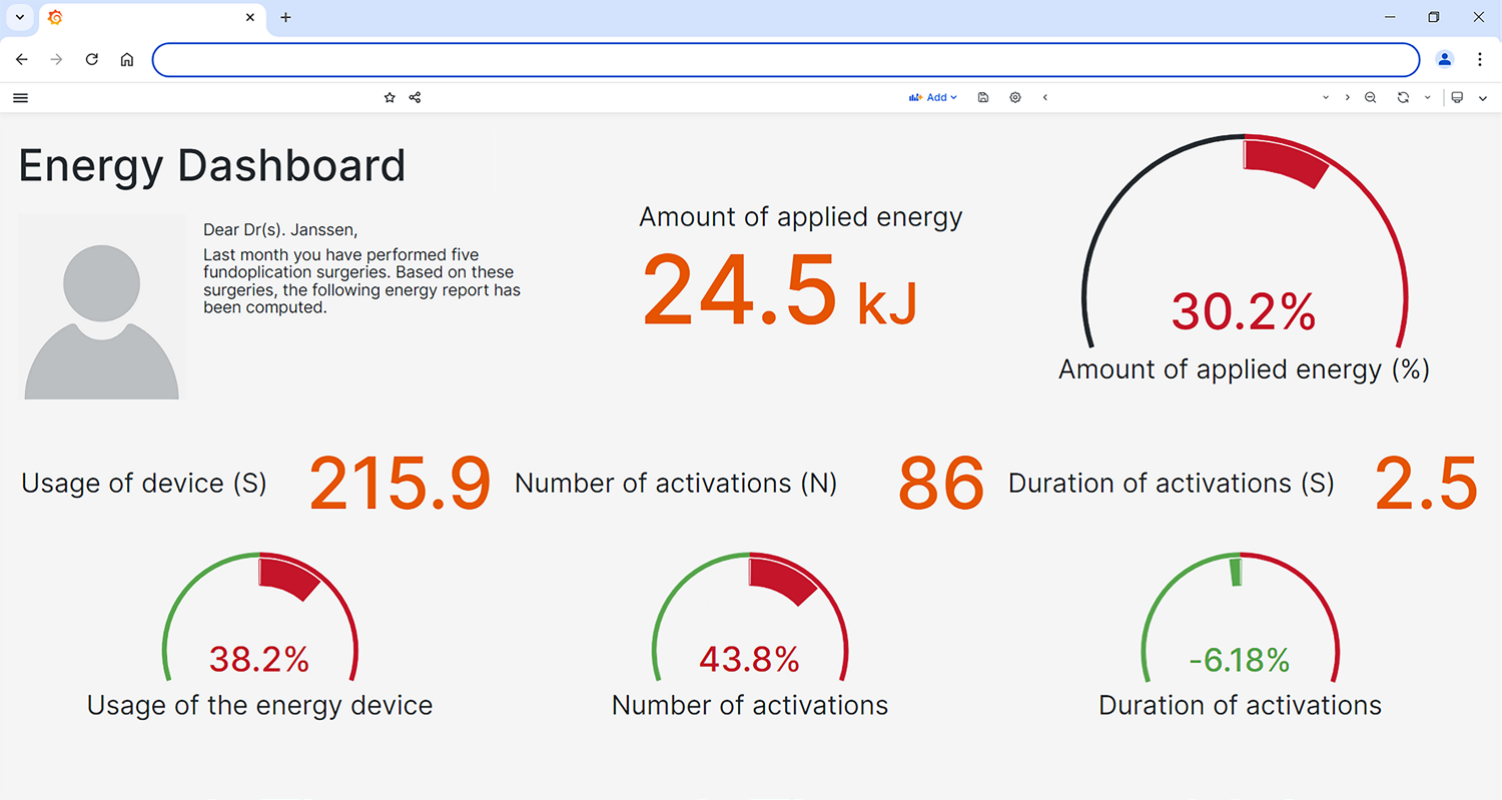
An example of how the energy device dashboard summarizes the data provided by the energy generator and the laparoscopic videos, enabling the comparison of applications

A visualization of the personalized energy dashboard is given in Fig. 4. The online dashboard displays personal metrics in absolute value and in comparison with other specialists. The comparison of the proposed methodology with the manually annotated tool activations showed an F1-score of 0.95.

### Pilot study

**Fig. 5:**
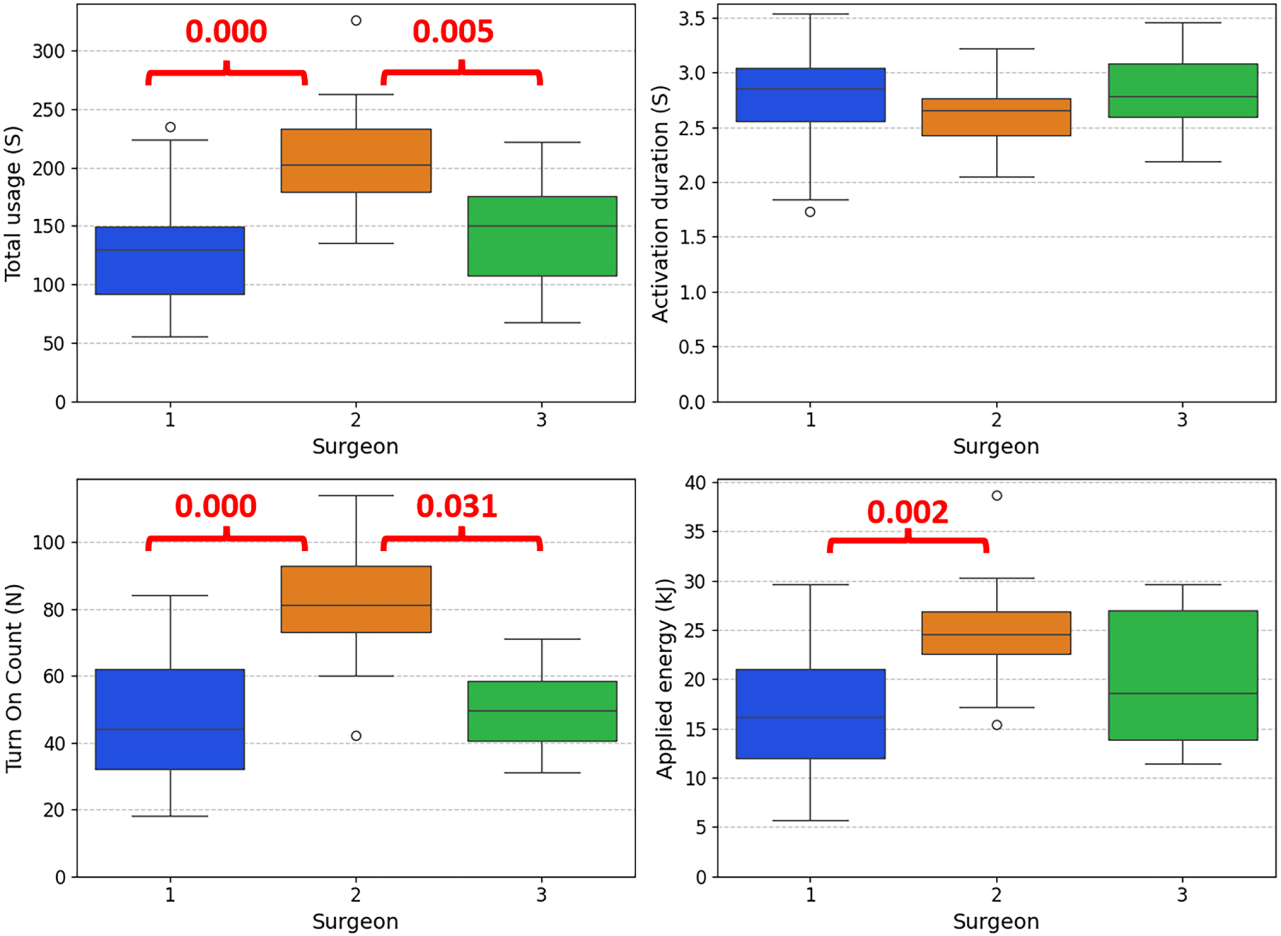
Distribution of the four metrics across three surgeons. The boxplot shows the median, interquartile range, and outliers. The *P* values of the significant differences by the ANOVA test followed by Tukey’s HSD test (*P* < 0.05) are shown in red (Color figure online)

This study included forty-eight patients from which both video and energy generator data were gathered. Surgeon 1 performed twenty-five procedures, Surgeon 2 did thirteen, and Surgeon 3 did nine. Table 1 summarizes the distributions of the metrics about surgical device use. A comparison between the three surgeons for the different metrics is also illustrated in Fig. 5. The box plot shows a higher total use, turn-on count, and amount of applied energy for Surgeon 2 than for Surgeon 1 and 3. These differences were statistically significant for the total use and turn-on count (One-way ANOVA test followed by Tukey’s HSD test, *P* < 0.05). The applied energy only differed significantly between Surgeon 1 and Surgeon 2.

**Table 1:** The mean and standard deviation of the metrics on surgical device use by three surgeons with different levels of expertise

Surgeon	1	2	3
Total use time (s)	130.3 ± 44.8	204.3 ± 51.9	144.6 ± 51.3
Turn on count (n)	48.9 ± 18.9	80.2 ± 19.6	53.1 ± 15.7
Activation duration (s)	2.8 ± 0.4	2.6 ± 0.3	2.7 ± 0.5
Applied energy (kJ)	16.7 ± 6.5	24.4 ± 5.9	19.6 ± 7.3

**Fig. 6:**
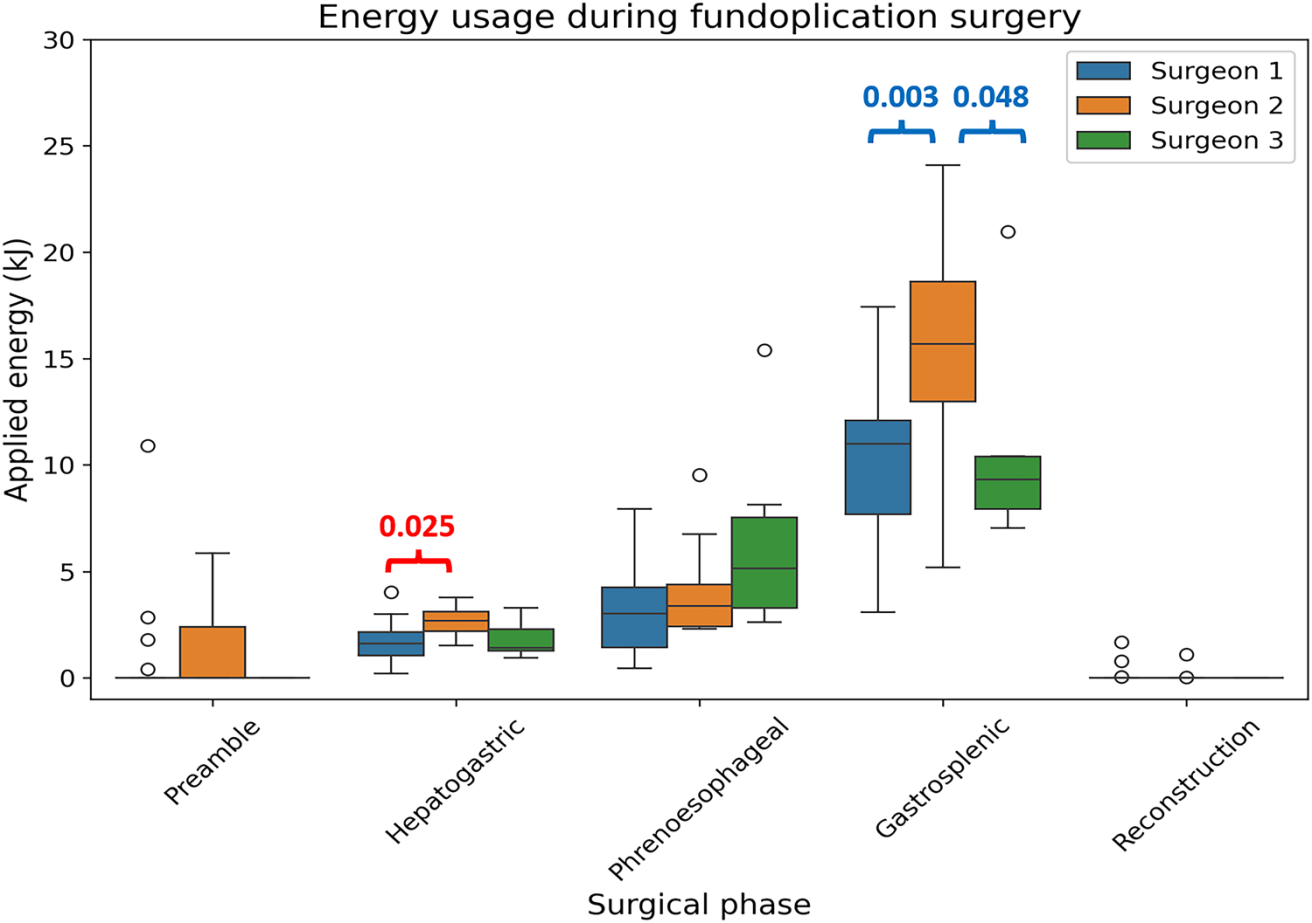
The applied energy (kJ) over the five phases of the fundoplication surgery. The boxplot shows the median, interquartile range, and outliers. The *P* values of the significant differences by the ANOVA test followed by Tukey’s HSD test (*P* < 0.05) are shown in red. The blue values indicate the *P* values of the significant differences by the Kruskal–Wallis test followed by the Dunn’s test (*P* < 0.05) (Color figure online)

Figure 6 and Table 2 provide an overview of the applied energy over the phases of the fundoplication surgery. These results show that most energy is applied in the gastrosplenic phase of the surgery. There was a significant difference (One-way ANOVA test followed by Tukey’s HSD test, *P* < 0.05) in applied energy between Surgeon 1 and 2 for the hepatogastric phase. The applied energy during the gastrosplenic phase differed significantly (Kruskal–Wallis followed by Dunn’s test, *P* < 0.05) between the different surgeons (1 vs. 2 and 2 vs. 3). There were no significant differences for the other surgical phases.

**Table 2:** The mean and standard deviation of the applied energy (kJ) by the surgical device use of three surgeons with different levels of expertise

Surgeon	1	2	3
1. Preamble	0.6 ± 2.2	1.3 ± 2.2	0.0 ± 0.0
2. Hepatogastric ligament	1.6 ± 1.0	2.7 ± 0.7	1.8 ± 0.9
3. Phrenoesophageal membrane	3.2 ± 2.1	4.0 ± 2.1	6.6 ± 4.8
4. Gastrosplenic ligament	10.1 ± 3.9	15.8 ± 5.0	10.8 ± 5.1
5. Hiatal constriction	0.1 ± 0.4	0.1 ± 0.3	0.0 ± 0.0

## Discussion

In this study, we present a post-operative energy dashboard to teach surgeons about electrosurgical device use. We showed that the energy generator and laparoscopic video data can accurately be transformed into a post-operative report. In addition, the pilot study showed significant differences in the application by the different surgeons. Therefore, the study results indicated that the proposed methodology can provide new insights for surgical skills assessment.

The comparison in Fig. 1 shows the potential insights energy monitoring can add to the current surgical skills assessment. Personal scores should be compared to large datasets on comparable surgeries to obtain well-founded insights. Therefore, we plan to expand to a multi-center study with a larger sample of surgeons and surgical procedures. For this, the platform must be deployed on a large scale. The proposed method of obtaining data on the application of electrosurgery is independent of the type of diathermy or the type of procedure, which offers the opportunity to expand the use of the energy dashboard easily. However, several post-processing and manual steps are still required to acquire the knowledge. A direct, automated data integration from the energy generator and the laparoscopic video would facilitate the broader dashboard application. Ultimately, it should be investigated whether the energy dashboard and this novel form of skills assessment led to an improved application of electrosurgery, which means less energy while maintaining adequate hemostasis and dissection. Clinical studies will have to determine the ideal energy use for effective hemostasis with minimal tissue coagulation. This future work will correlate energy metrics with hemostasis quality to establish optimal energy use.

Assessing the application throughout the different procedural phases provided further in-depth insight into this dynamic use. Figure 6 showed how much energy device use differs between the surgical phases. This distinction showed that the large differences in the total applied energy of Surgeon 2 with respect to Surgeon 1 and 3 (Table 1) mainly resulted from its application in the gastrosplenic phase. In addition, the applied energy in the preamble and reconstruction phase resulting from adhesiolysis could be distinguished from the energy applied in other surgical phases. These insights highlight the importance of integrating the video and energy data sources. In certain surgical phases, it may be preferable to provide less energy as minimizing thermal spread to neighboring tissues could be more important than ensuring perfect hemostasis, e.g., in the vicinity of Vagal nerve branches. Therefore, surgeons should adopt a dynamic approach when using a sealing tool. A phase-level evaluation of the device application enabled the assessment of this dynamic approach. In the next phase, we aim to automate this analysis by computer vision-based surgical phase detection [12].

In addition to monitoring electrosurgery use, we have developed algorithms to quantify blood loss as an indicator for the effectiveness of hemostasis. The objective of the dashboard is to perform surgery with minimal energy and tissue coagulation while maintaining optimal hemostasis. Therefore, the extent to which the activations of the device lead to bleeding should also be quantified in the dashboard. Device-induced bleeding should be identified by linking the activation moments in the generator data to the onset of bleeding in the laparoscopic video. Our current work focuses on AI-based blood detection algorithms near device activation on the timeline of generator-derived tool activation information [13, 14].

Future studies could focus on the broader application of the energy dashboard. This study only focused on a vessel sealing device with fixed generator settings, but electrosurgical devices with variable settings (e.g., surgical pencils) could also be assessed. The methodology presented can be adapted to acquire the relevant metrics of this device type. Generator settings (e.g., coagulate vs. cutting mode) can be derived based on the manufacturer to enable a more detailed analysis of electrosurgical practices. In addition, the current study provides insights into energy use postoperatively but does not offer real-time feedback. Future studies could potentially evaluate the impact of real-time dashboard feedback on energy use, although we need to consider the generalizability of the feedback for individual cases.

## Conclusion

This study demonstrated that monitoring energy use during surgery can highlight differences in how electrosurgical devices are applied in the operating room. Additionally, the pilot study revealed significant variations in the use of electrosurgical devices among different surgeons. By expanding the application of the energy dashboard, we can create a valuable new source of information for surgical skills assessment.
